# Yishen Qingli Heluo Granule Ameliorates Renal Dysfunction in 5/6 Nephrectomized Rats by Targeting Gut Microbiota and Intestinal Barrier Integrity

**DOI:** 10.3389/fphar.2022.858881

**Published:** 2022-06-22

**Authors:** Xian Sun, Jie Chen, Yiting Huang, Sha Zhu, Shuaishuai Wang, Zijing Xu, Junfeng Zhang, Wei Sun

**Affiliations:** ^1^ The First Clinical Medical College, Nanjing University of Chinese Medicine, Nanjing, China; ^2^ Hanlin College, Nanjing University of Chinese Medicine, Nanjing, China; ^3^ School of Medicine and Holistic Integrative Medicine, Nanjing University of Chinese Medicine, Nanjing, China; ^4^ Department of Nephrology, Jiangsu Province Hospital of Chinese Medicine, Affiliated Hospital of Nanjing University of Chinese Medicine, Nanjing, China

**Keywords:** chronic kidney disease, yishen qingli heluo granule, gut microbiota, intestinal barrier, microbial translocation

## Abstract

Chronic kidney disease (CKD) is often accompanied with imbalanced gut microbiota and impaired intestinal barrier. Hence, efforts to ameliorate renal dysfunction by manipulating gut microbial ecosystem are underway. Yishen Qingli Heluo granule (YQHG) is a representative traditional Chinese medicine (TCM) prescription for clinical treatment of CKD. However, its underlying mechanism has not been well elucidated. This study aimed to explore effects of YQHG on renal dysfunction in 5/6 nephrectomized rats by targeting gut microbiota and intestinal barrier. Here, we found that YQHG provided significant renal protection in 5/6 nephrectomized rats by reducing renal fibrosis and inflammation, reestablishing bacterial communities, and improving intestinal barrier. Our analysis showed that YQHG altered the bacterial community of 5/6 nephrectomized rats. In particular, the prescription significantly increased the relative abundance of SCFA-producing bacteria (i.e., Lactobacillaceae, Lactobacillus and Lactobacillus_gasseri), which was contributed to the improved SCFA concentration (i.e., total SCFA, acetic acid, butyric acid) and intestinal barrier (i.e., the improved permeability and microbial translocation). More critically, microbiota-transfer study showed that the protective effect of YQHG was partly attributed to the mediation of the gut microbiota, especially the SCFA-producing bacteria. Our current findings propose a microbiota-targeted intervention and indicate that YQHG may become a novel promising treatment for CKD.

## Introduction

Chronic kidney disease (CKD) is defined as the sustained presence of abnormalities of kidney structure or function. It is a major public health burden, affecting more than 8% of the population worldwide (GBD Chronic Kidney Disease Collaboration, 2020). At present, the number of people receiving renal replacement therapy has exceeded 2.6 million and is expected to double to 5.4 million worldwide by 2030 (Liyanage et al., 2015). Once CKD progresses to ESRD, it will not only bring economic burden to patients, but also affect their quality of life to a great extent. However, the risk factors and disease pathways that lead to compromised renal function are diverse and poorly clarification. Consequently, few strategies are available to delay the progression of CKD, with most current treatment strategies centred around reducing proteinuria, lowering blood pressure and uric acid (Abboud and Henrich, 2010). Clearly, additional therapeutic avenues are needed to manage, control and ultimately alleviate CKD.

Advances in the metagenomic studies and the novel computational tools, combined with the mechanistic studies in disease models, are accelerating our understanding of the importance of gut microbiota in health and disease (Erny et al., 2021; Wilkinson et al., 2021). Of note, research in recent years has linked the alteration of gut microbiota, a condition termed dysbiosis, with chronic disease outside the digestive system such as CKD (Vaziri et al., 2013a; Jiang et al., 2017). Gut microbiota dysbiosis and impaired intestinal barrier are the most important causative factors of crosstalk between the gut microbial ecosystem and CKD. In short, CKD-related changes in the gut microbiota led to the impaired intestinal barrier and the microbial translocation. Consequently, the influx of bacterial components into the kidney via the systemic circulation contributes to renal inflammation (Anders et al., 2013; Andersen et al., 2017). Therefore, correcting this dysbiosis or conducting the specific microbiota-targeted interventions may become potential strategies for the prevention and management of CKD (Ramezani and Raj, 2014). Short-chain fatty acids (SCFAs) are a group of gut-microbiota-derived lipid metabolites, which play an important regulatory role in maintaining the integrity of the intestinal barrier (Liu et al., 2021). Compared with healthy controls, a decrease in the abundance of the SCFA-producing bacteria was observed in CKD patients, which in turn led to a lack of SCFAs (Wong et al., 2014). An emerging evidence indicated that the concentrations of SCFAs, especially acetic acid and butyric acid, were almost completely suppressed in humans and animal models with CKD (Jiang et al., 2016; Mishima et al., 2017). In addition, an increasing body of evidence demonstrated the reduction of SCFAs could compromise the renal function (Steenbeke et al., 2021). Conversely, supplementation of SCFAs, particularly butyric acid, could promote barrier function and control microbial translocation, and ultimately protect the kidney from injury (Wang et al., 2019). In summary, targeting the gut microbiota, especially the SCFA-producing bacteria, may provide a promising treatment for CKD.

Yishen Qingli Heluo granule (YQHG) was independently developed by Jiangsu Province Hospital of Chinese Medicine as a TCM prescription for the clinical treatment of CKD. The clinical studies had demonstrated that YQHG could improve the clinical symptoms and reduce the level of serum creatinine (Scr). Moreover, the progression of CKD3 to CKD4 could also be delayed by YQHG (Zhao et al., 2017). In addition, previous studies had shown that some TCM herbs or their active components in YQHG could increase the SCFA concentrations and promote intestinal barrier function. For instance, a new type of polysaccharide (AERP) extracted from Astragalus membranaceus improved the butyric acid concentration and the gut microbiota dysbiosis (Liu et al., 2019). Moreover, the latest study found that Rhubarb could alleviate the progress of CKD by remodeling the gut microbiota and repairing the intestinal barrier (Ji et al., 2020). In summary, YQHG has a therapeutic effect on CKD, and part of its mechanism may be attributed to the improved gut microbiota dysbiosis and intestinal barrier impairment. Therefore, based on the gut microbial ecosystem, our study explored the mechanism of YQHG alleviating renal dysfunction in 5/6 nephrectomized rats by targeting SCFA-producing bacteria and the intestinal barrier.

## Materials and Methods

### Preparation and Testing of Experimental Drugs

YQHG (batch number 20051031) was composed of 10 single TCM granules: Angelicae Sinensis Radix (Danggui, DG), Achyranthis Bidentatae Radix (Niuxi, NX), Centella Asiatica (L.) Urban (Jixuecao, JXC), Polygonati Rhizoma (Huangjing, HJ), Smilacis Glabrae Rhixoma (Tufuling, TFL), Radix Rhei Et Rhizome (Dahuang, DH), Pyrrosiae Folium (Shiwei, SW), Hedysarum Multijugum Maxim (Huangqi, HQ), Serissa Japonica (Thunb.) Thunb (Liuyuexue, LYX), Polygoni Cuspidati Rhizoma Et Radix (Huzhang, HZ), which was manufactured by Jiangyin Tianjiang Pharmaceutical Co., Ltd. (Wuxi, Jiangsu, China). The ratios of the single TCM granule to the corresponding crude drug were listed in Table 1. The 10 TCM granules of YQHG were authenticated by Professor Wei Sun (Nanjing University of Chinese Medicine, Nanjing, Jiangsu, China). Their voucher specimens were deposited at Jiangsu Province Hospital of Chinese Medicine, Affiliated Hospital of Nanjing University of Chinese Medicine. The weight-based drug dosing format (per kg) was used in this study to calculate the YQHG doses in rats (Sharkey et al., 2001). Calculated with an adult body weight of 70 kg, the clinical equivalent dose converted into rats was 2.8 g/kg. Based on previous studies, the appropriate oral gavage dose in our study was twice the clinical equivalent dose of YQHG, which was 5.6 g/kg per day. UHPLC-MS was conducted to confirm the stability and quality of YQHG extract.

**TABLE 1:** Details of YQHG.

Chinese name	Latin name	Phylum	Family	Genus	Parts used	Amount used (g)	Ratio
Danggui	Angelicae sinensis radix	Angiospermae	Umbelliferae	Angelica L.	Root	4	2:5
Niuxi	Achyranthis bidentatae radix	Angiospermae	Amaranthaceae	Achyranthes L.	Root	3	3:10
Jixuecao	Centella asiatica (L.) urban	Angiospermae	Umbelliferae	Centella L.	Herba	3	1:10
Huangjing	Polygonati rhizoma	Angiospermae	Liliaceae Juss.	Polygonatum Mill.	Rhizome	8	2:5
Tufuling	smilacis glabrae Rhixoma	Angiospermae	Liliaceae	Smilax L.	Rhizome	2	1:15
Dahuang	Radix rhei et rhizome	Angiospermae	Polygonaceae	Rheum L.	Rhizome	2	1:3
Shiwei	Pyrrosiae folium	Pteridophyta	Polypodiaceae	Pyrrosia Mirbel.	Leave	2	1:10
Huangqi	Hedysarum multijugum Maxim	Angiospermae	Leguminosae sp.	Astragalus Linn.	Root	4.5	3:20
Liuyuexue	Serissa japonica (Thunb.) thunb	Angiospermae	Rubiaceae Juss.	Serissa Comm. ex Juss.	Whole plant	1	1:30
Huzhang	Polygoni cuspidati rhizoma et radix	Angiospermae	Polygonaceae	Reynoutria Houtt.	Rhizome, Root	1	1:15

### 5/6 Nephrectomy and Drug Intervention Regimen

Male Sprague Dawley (SD) rats (no. 20210324Aazz0619000891, 6–8 weeks old, 200–230 g) were provided by Vital River Experimental Animal Co., Ltd. (Zhejiang, China). Rats were housed in a specific pathogen-free experimental animal center of Nanjing Agricultural University. Specifically, rats were housed in plastic cages and were freely provided with sufficient standard food and water. The environmental conditions of the room where the rats were raised included temperature (22 ± 2°C), humidity (50% ± 10%), and 12/12 h light/dark cycle. All animal experiments were performed according to the protocol approved by Animal Care and Use Committee of Nanjing Agricultural University (permission number PZW20200013). All surgeries were performed under appropriate anesthesia, and every efforts were made to minimize animal suffering.

**FIGURE 1:**
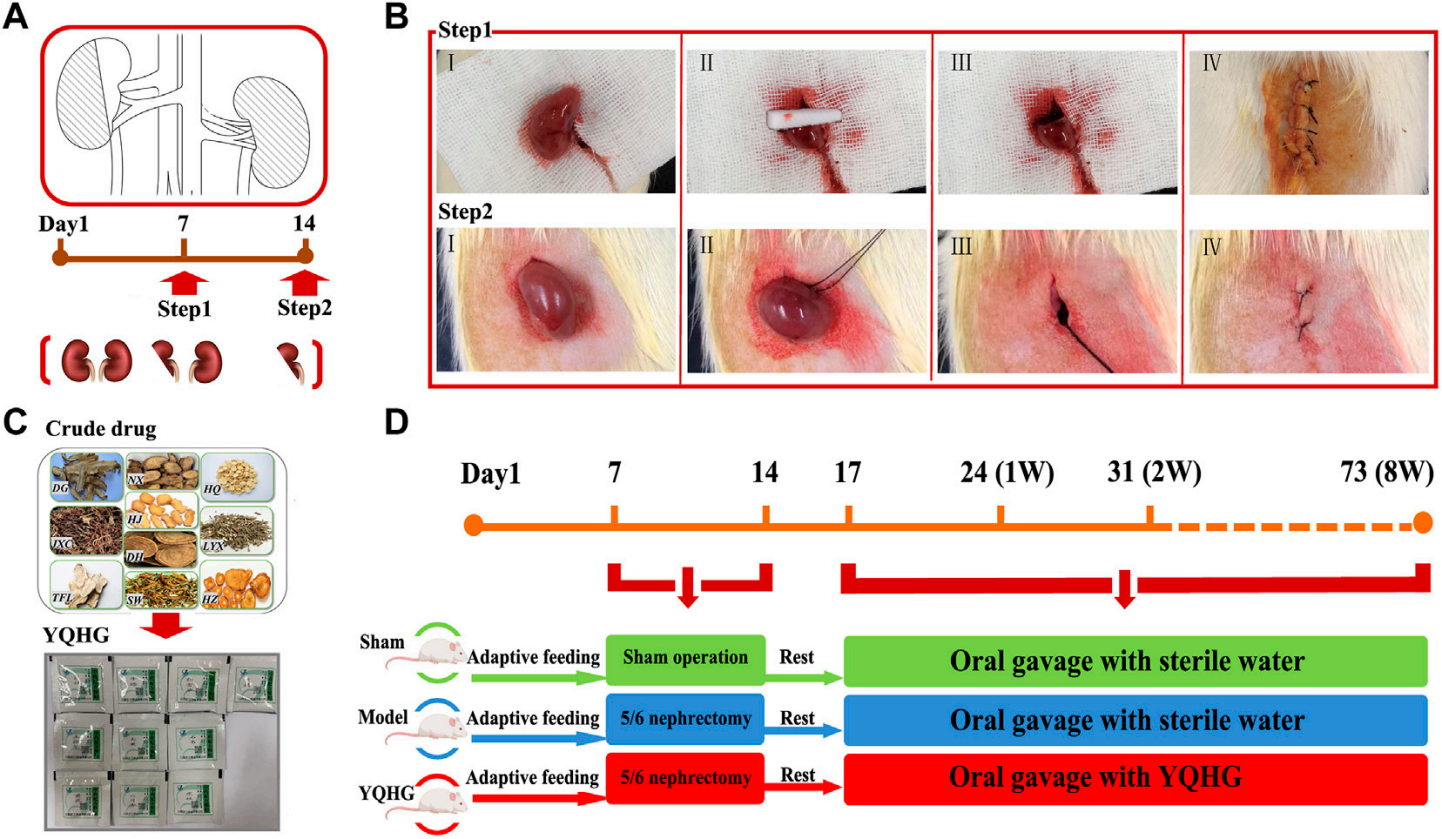
The 5/6 nephrectomy and drug intervention regimen. **(A)** Pattern diagram of 5/6 nephrectomy. **(B)** Surgical diagram of 5/6 nephrectomy. Step1: ablated 2/3 of the left kidney. Ⅰ Exposed the left kidney. Ⅱ Removed 2/3 of the left kidney and compressed the wound with gelatin sponge to stop bleeding. Ⅲ Confirmed that there was no bleeding on the cut surface, and then put the residual kidney into the abdominal cavity. Ⅳ Sutured the muscle layer and skin layer by layer, and applied iodophor for disinfection. Step2: nephrectomy of the right kidney. Ⅰ Exposed the right kidney. Ⅱ Ligation of renal pedicle with a sterile 3–0 suture. Ⅲ Removal of the right kidney. Ⅳ Sutured the muscle layer and skin layer by layer, and applied iodophor for disinfection. **(C)** Ten single granules of YQHG. **(D)** Time axis of model construction and drug intervention.

CKD was induced by a two-step 5/6 nephrectomy as previously described (Zhang H.-F. et al., 2019). The procedure of the two-step operation was shown in Figures 1A,B. The operation process followed the principle of sterility. Notably, the abdominal cavity was flushed with 0.9% sterile saline to avoid postoperative abdominal adhesion after two surgical operations. Moreover, induction and maintenance of anesthesia (consumption of Isoflurane was 6 ml/h and 3 ml/h, respectively) during the operation were carried out with the Rodent Gas Anesthesia Machine (Yuyan-ABS). The sham-operated rats were stripped of fat sac without nephrectomy, while the surgical anesthesia and two operation time nodes were consistent with the 5/6 nephrectomized rats. In this experiment, rats underwent sham operation were used as sham group. The rats with 5/6 nephrectomy were randomly divided into model group and YQHG group. The daily doses of gavage substances were as follows: 1) sham group (sterile water); 2) model group (sterile water); 3) YQHG group (5.6 g/kg YQHG) (Figures 1C,D). Detailed grouping and drug intervention regimen will be further elaborated. During the experiment, all rats were weighed twice a week, and the drug doses were adjusted accordingly. At the end of the experiment, urine and fecal samples were collected from rats before sacrificed. Blood samples, renal and colon tissues were collected for further studies.

### Co-Housing and Fecal Microbiota Transplantation Regimen

Recent works suggested that gut microbiota would be transferred between rodents in a shared living environment (i.e., co-housing). Specifically, the co-housing strategy is based on the fecal-eating characteristics of rodents. Co-housing of rodents with different microbiota would generate hybrid-microbiota (intermediate phenotype), which indicated that was a two-way microbial transfer (Surana and Kasper, 2017; Ralston et al., 2021). Co-housing and fecal microbiota transplantation (FMT) were the preferred strategies to achieve microbial transfer, which were widely used in the studies of causal relationship between gut microbiota and disease (Wu et al., 2019; Cai et al., 2021; Zhao et al., 2021). Based on the pre-experiment of microbiota transfer, in order to achieve the best effect of microbiota transfer, we chose co-housing combined with FMT in this study. Based on previous studies (Ji et al., 2019; Hong et al., 2021), the time for co-housing and FMT was set to 3 weeks in our study to achieve the microbial transfer, and to maximize the control of changes (such as physiological, gut microbiota) caused by time factors. The specific co-housing implementation regimen would be further illustrated through the flow chart in combination with the experimental grouping.

FMT was performed based on an established protocol (Ma et al., 2021; Sun et al., 2021). Briefly, fresh feces from the rats with YQHG treatment were collected under sterile conditions. Every 100 mg mixed feces were re-suspended in 1 ml of sterile saline. The solution was vigorously vortexed for 10 s before centrifugation at 1000 g (4°C) for 3 min. The suspension was collected and used as transplant material. Fresh transplant material was prepared within 10 min before oral gavage on the day of transplantation to prevent changes in the composition of the gut microbiota. The recipient rats were fed with fresh transplant material (1 ml for each rat daily) by oral gavage for 3 weeks.

### Scr, BUN, and 24 h Urinary Protein Assay

Scr, BUN and 24 h urinary protein levels were measured using Scr, BUN and urinary protein quantification assay kits (C011-2-1, C013-2-1, C035-2-1; Nanjing Jiancheng Biotech Co., Ltd.) according to the manufacturer’s instruction. Among them, urinary protein quantification was undertook by 24 h urine collection with metabolic cages on the day before the rats were sacrificed. The formula is as follows: Urinary protein (mg/24 h) = urinary protein concentration (mg/L) × 24 h urine volume (L).

### Histopathologic Evaluation and Fluorescence *in Situ* Hybridization

Tissue samples (kidney and colon) of rats were rinsed with precooled PBS (Biosharp) and then fixed in 4% paraformaldehyde (biosharp). Sections (4 μm) were cut from paraffin-embedded tissues and stained with H&E or Masson’s trichrome (Masson) (Solarbio). Histological images were captured using a Digital Slide Scanner (Type specification: Pannoramic DESK, P-MIDI, P250; Company: 3D HISTECH; Country: Hungary; Scanning software: Pannoramic Scanner). The steps for calculating the area of glomerular and tubulointerstitial fibrosis were as follows: First of all, CaseViewer2.4 software was used to select the glomerular (tubulointerstital) area for 40 × imaging. Try to fill the entire field of view with the tissue, and ensure that the background light of each image was consistent. Secondly, Image-Pro Plus 6.0 software (Media Cybernetics, Inc., United States) performed image analysis, set pixel as the standard unit, and two fields of view were selected for each slice. Finally, the fibrosis area was evaluated, that was, the percentage of fibrosis area (%) = glomerular (tubulointerstital) fibrosis area/glomerular (tubulointerstital) area × 100%.

Fluorescence *in situ* hybridization (FISH) is an effective approach for locating the spatial distribution of bacteria in tissue samples (Wang et al., 2020). In our study, FISH analysis was performed on renal tissues of rats, using Digoxin (DIG)-labeled universal probes targeting bacterial 16 S rRNA gene (Lim and Lim, 2017). FISH staining was performed as previously described (Moter and Göbel, 2000). The fluorescence signals were observed and detected by fluorescence microscope. The images were captured at × 200 magnification and processed by Image-Pro Plus 6.0 software.

### Gut Microbiota Analysis

Feces of all rats were collected for gut microbiota analyses. The four main steps involved in the sequencing of gut microbiota were as follows: 1) Extraction of genome DNA: The total DNA of feces were extracted by E.Z.N.A.^®^ Soil DNA Kit (Omega Bio-tek) according to manufacturer’s protocols. DNA concentration and purity were monitored on 1% agarose gel. 2) PCR amplification: The V3-V4 region of 16 S rRNA gene was amplified using specific primer (338F_806R) with the barcode. The PCR amplification was carried out using TransStart Fastpfu DNA polymerase kit. 3) PCR products quantification, mixing and purification: Refer to the preliminary quantification results of electrophoresis, QuantiFluor™ -ST Assay Kit (Promega) was used to detect and quantify the PCR products, and then mix the corresponding proportions according to the sequencing requirements of each sample. Then, the mixture of PCR products was purified with GeneJET Gel Extraction Kit (Thermo Fisher Scientific). 4) Library preparation and sequencing: The profiling was carried out on the MiSeq platform (Illumina, Inc., San Diego, CA, United States). The 16 S rRNA sequencing data were quality filtered using Fast Length Adjustment of Short reads (FLASH, Version 1.2.11). Operational taxonomic units (OTUs) were picked at a 97% similarity cut-off, and the purified amplicons were sequenced on an Illumina MiSeq platform at Majorbio Biopharm Technology Co., Ltd. (Shanghai, China) according to the standard protocols.

### Fecal Short-Chain Fatty Acid Concentration Determination

50 μl of 15% phosphoric acid, 100 μl internal standard (isohexanoic acid, 125 μg/ml) solution, 400 μl of ether were added in an appropriate amount of fecal sample from rats; The Sample was homogenized for 60 s at 60 Hz (Repeated 2 times), and centrifuged at 12,000 rpm (4°C) for 10 min. The supernatant was collected and stored at −20°C until further processing. Concentrations of SCFAs were quantified by Gas Chromatography-Mass Spectrometry (GC-MS) (GC: Thermo, TRACE 1310; MS: Thermo, ISQ LT) detection method according to established protocols (Hsu et al., 2019; Zhang S. et al., 2019).

### Intestinal Permeability Determination

FITC-dextran (FD-4, 4kDa, Sigma-Aldrich Corp., United States) was used to confirm gut leakiness *in vivo*. FITC-dextran was dissolved in PBS at a concentration of 100 mg/ml and administered to each rat (44 mg/100 g body weight) by oral gavage. A gap of 30 min between each rat was recommended for the FITC-dextran oral gavage (Gupta and Nebreda, 2014). After 4 h, the blood was collected. The blood was mixed by inverting the vacuum SST tube 3–4 times, and stored at 4°C in the dark. SST tubes were processed to separate the serum after blood had been collected from all the rats. Diluted the serum with an equal volume of PBS and added 100 μl of diluted serum to a 96-well microplate in duplicate. The concentration of FITC-dextran in serum was determined by spectrophotofluorometry with an excitation wavelength of 485 nm (20 nm band width) and an emission wavelength of 528 nm (20 nm band width). The serially diluted FITC-dextran (0, 125, 250, 500, 1,000, 2,000, 4,000, 6,000, 8,000 ng/ml) was used for the subsequent standard curve drawing. Serum from rat not administered with FITC-dextran was used to determine the background.

### ELISA

PBS was added to the renal tissue (1 ml/0.1 g), and electro-homogenized sample was centrifuged to collect the supernatant. Subsequently, the rat IL-6 ELISA kit (bsk00042, Nanjing Jinyibai Biotech Co., Ltd.) was used to detect the expression of IL-6 according to the manufacture’s instructions.

### Statistical Analysis

All data were expressed as means ± SEM. GraphPad Prism 9.0 software (GraphPad, CA, United States) was used for statistical analysis and image construction. We combined QQ plots with the Shapiro-Wilk test to assess data normality. For normally distributed data, one-way ANOVA followed by Tukey’s test was used. For non-normally distributed data, Kruskal-Wallis test followed by non-parametric Wilcoxon rank-sum test was used. *p* < 0.05 was considered statistically significant.

## Results

### Standardization of YQHG

Before drug treatment, YQHG was chemically standardized. An UHPLC-MS approach was established to reveal the stability and qualification of YQHG (Supplementary Figure S1). The analytical conditions and sample preparation were as follows:

1) Analytical conditions: ①LC-MS/MS analysis was performed on a Agilent ultra-high performance liquid chromatography 1290 UPLC system with a Waters UPLC BEH C18 column (1.7 μm 2.1*100 mm). The flow rate was set at 0.4 ml/min and the sample injection volume was set at 5 μl. The mobile phase consisted of 0.1% formic acid in water (A) and 0.1% formic acid in acetonitrile (B). The multi-step linear elution gradient program was as follows: 0–3.5 min, 95%–85% A; 3.5–6 min, 85%–70% A; 6–6 5 min, 70%–70% A; 6.5–12 min, 70%–30% A; 12–12.5 min, 30%–30% A; 12.5–18 min, 30%–0% A; 18–25 min, 0%–0 % A; 25–26 min, 0%–95% A; 26–30 min, 95%–95% A. ②An Q Exactive Focus mass spectrometer coupled with an Xcalibur software was employed to obtain the MS and MS/MS data based on the IDA acquisition mode. During each acquisition cycle, the mass range was from 100 to 1,500, and the top three of every cycle were screened and the corresponding MS/MS data were further acquired. Sheath gas flow rate: 45 Arb, Aux gas flow rate: 15 Arb, Capillary temperature: 400°C, Full ms resolution: 70,000, MS/MS resolution: 17,500, Collision energy: 15/30/45 in NCE mode, Spray Voltage: 4.0 kV (positive) or -3.6 kV (negative).
2) Sample preparation: the samples were thawed on ice. After 30 s vortex, the samples were centrifuged at 12,000 rpm (RCF = 13,800 (×g), R = 8.6 cm) for 15 min at 4°C. 300 μl of supernatant was transferred to a fresh tube and 1,000 μl of extracted solution containing 10 μg/ml of internal standard was added, then the samples were sonicated for 5 min in ice-water bath.After placing 1 h in −40°C, the samples were centrifuged at 12,000 rpm (RCF = 13,800 (×g), R = 8.6 cm) for 15 min at 4°C. The supernatant was carefully filtered through a 0.22 μm microporous membrane, then take 100 μl from each sample and pooling as QC samples. Store at −80°C until the UHPLC- MS analysis.

YQHG is a representative traditional Chinese medicine (TCM) remedy for clinical treatment of CKD. However, its underlying mechanism has not been well elucidated. Our preliminary research conducted the disease target prediction of YQHG through network pharmacology. We found that these six components (Quercetin, Kaempferol, Isorhamnetin, Formononetin, Luteolin, Emodin) could be matched to therapeutic targets and were the main therapeutic components in YQHG. In addition, TCM mainly treats diseases at the system level. Whether other components also have the therapeutic effect needs to be further explored, which is also the direction of our later research. Non-target metabolomics method was used in this study, which detected relative abundance (peak area) of individual components. Relative abundance (peak area) of the six components were shown in Supplementary Table S1.

The peak positions of Quercetin, Kaempferol on the total ion chromatogram were shown in Supplementary Figure S1. Because the total ion chromatogram reflects the ionic strength information of all metabolites in the sample, and the retention times of the two components are close, what we see from this graph is overlapping. Therefore, we extracted and amplified the peak graph for the two components to make it easier to distinguish the two. In addition, we also supplemented the secondary mass spectrometry information of the two components to prove the difference between the two (Supplementary Figure S2).

In summary, YQHG could be used as qualified sample for the following animal study.

### YQHG Improved Renal Function and Fibrosis in 5/6 Nephrectomized Rats

**FIGURE 2:**
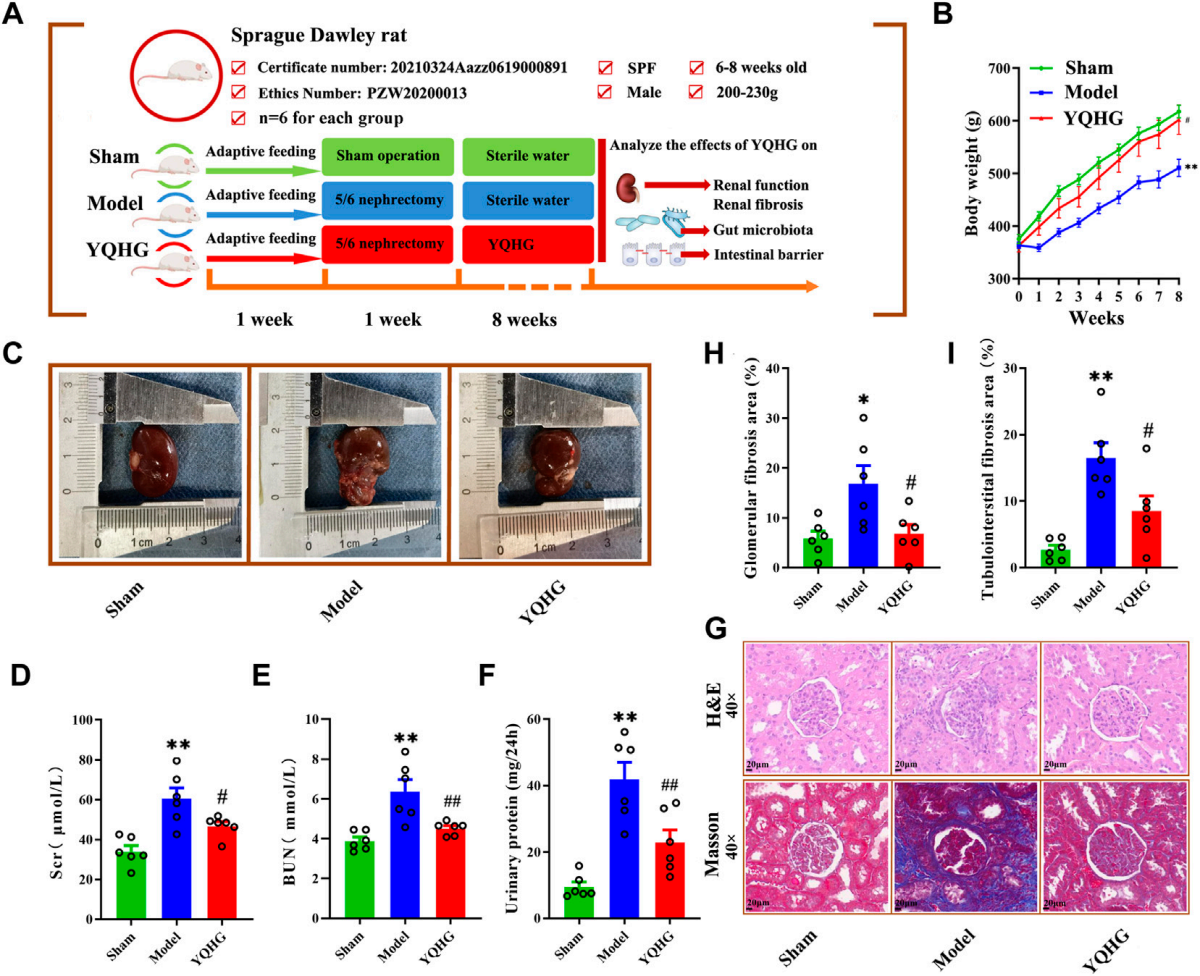
YQHG improves renal function and fibrosis in 5/6 nephrectomized rats. **(A)** Experimental design. **(B)** The body weight of the rats was measured. **(C)** The kidney of the rats was photographed. **(D–F)** Effects of YQHG treatment on levels of Scr, BUN, and urinary protein in 5/6 nephrectomized rats. **(G)** Representative images of H&E (40 × Magnification, Scale bar 20 μm) staining and Masson (40 × Magnification, scale bar 20 μm) staining of renal tissues. **(H–I)** Quantitative analysis of glomerular fibrosis area and tubulointerstital fibrosis area based on Masson staining. All data are expressed as means ± SEM. For normally distributed data (body weight, Scr, BUN, urinary protein, glomerular fibrosis area, tubulointerstital fibrosis area), one-way ANOVA followed by Tukey’s test was used. *p < 0.05, **p < 0.01 vs. the sham group; ^#^ *p* < 0.05, ^##^ *p* < 0.01 vs. the model group.

A comprehensive assessment of renal appearance (colour, capsule, border, weight), related parameters (Scr, BUN, urinary protein, glomerular fibrosis area, tubulointerstitial fibrosis area), and renal tissue histopathology (H&E, Masson) were conducted to explore the potential therapeutic value of YQHG on CKD. To this end, the experimental design was established (Figure 2A). In this part, there were three groups in total, namely the sham group, the model group, and the YQHG group (*n* = 6 for each group). As expected, the model group exhibited weight loss, pale kidneys, uneven borders, and hard-to-peel capsules. Remarkably, YQHG treatment impeded disease progression, which was shown by increased weight of rats (Figure 2B), recovery of reddish-brown kidneys, integrity of borders, and smoothness of the capsules (Figure 2C). Scr, BUN, and urinary protein are the key markers indicating the renal dysfunction. Our results showed that compared with the model group, the levels of the three parameters were significantly decreased in the YQHG group (Figures 2D–F). Histopathological analysis clearly showed inflammatory infiltration, mesangial expansion, tubular atrophy and dilation, glomerular sclerosis, and interstitial fibrosis in 5/6 nephrectomized rats, all of which were dramatically ameliorated by YQHG treatment (Figure 2G). Moreover, the areas of glomerular and tubulointerstitial fibrosis were significantly reduced in the YQHG group (Figures 2H,I). Taken together, these data indicated that YQHG had a profound protective effect on 5/6 nephrectomized rats, which was characterized by alleviating tissue damage, improving renal function and reducing renal fibrosis.

### YQHG Altered Bacterial Communities and Increased the Abundance of SCFA-Producing Bacteria in 5/6 Nephrectomized Rats

**FIGURE 3:**
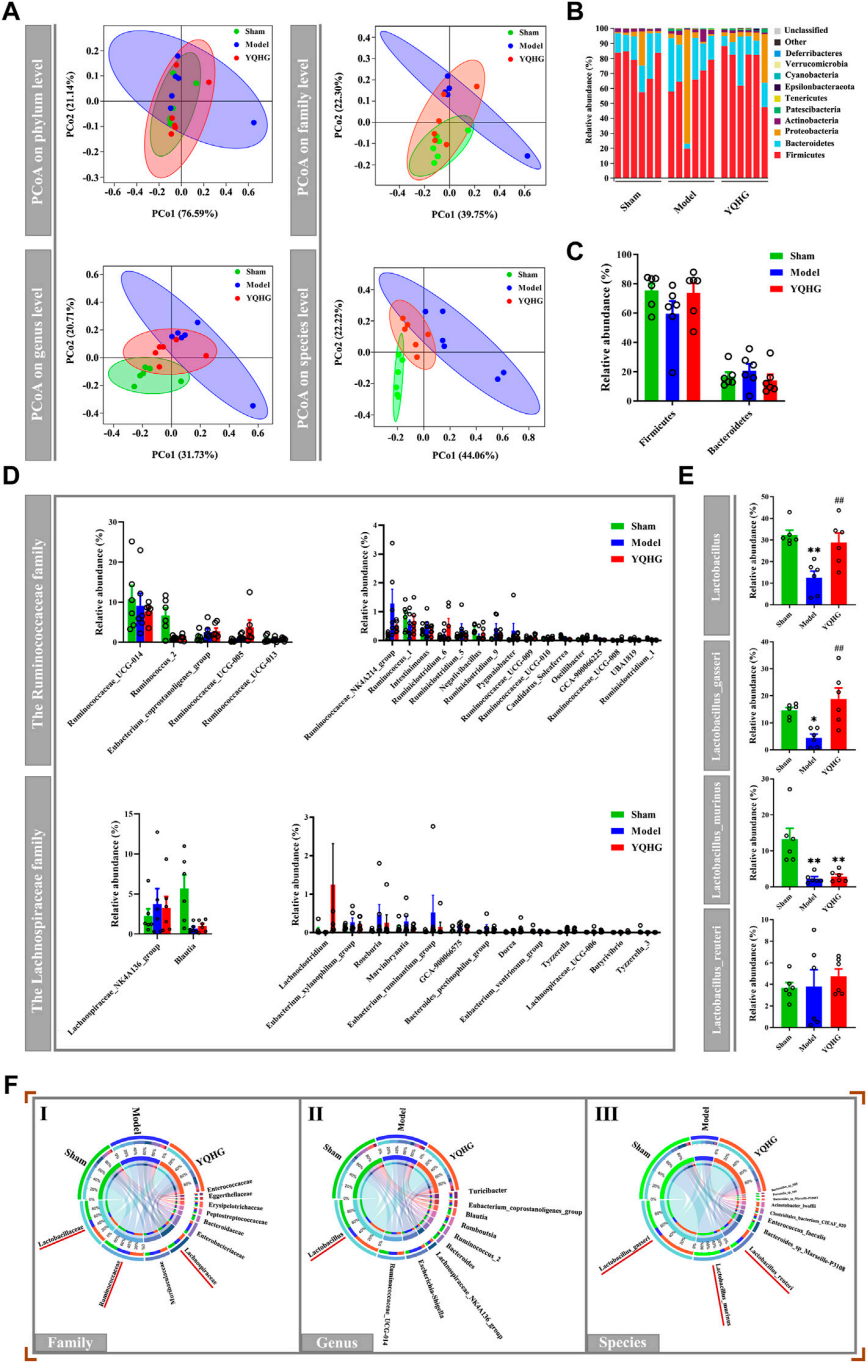
YQHG treatment modulates the gut microbiota in 5/6 nephrectomized rats. **(A)** PCoA based on Bray-Curtis distance in bacterial communities on four levels (phylum, family, genus, species). **(B)** Relative abundance of gut microbiota on phylum level. **(C)** Relative abundance of Firmicutes and Bacteroidetes on phylum level. **(D)** Relative abundance of genera within the Ruminococcaceae family and Lachnospiraceae family. **(E)** Relative abundance of genera and species within the Lactobacillaceae family. **(F)** Circos diagram of species-sample relationship on three levels (family, genus, species). The small semicircle (top half circle) indicates the composition of the gut microbiota in each group, the color of the outer ribbon represents which group it comes from, the color of the inner ribbon represents the gut microbiota, and the length represents the relative abundance of gut microbiota in the corresponding group; the large semicircle (bottom half circle) indicates the distribution ratio of gut microbiota in different groups on the taxa level. The outer color band represents the gut microbiota, the inner color band represents different groups, and the length represents the distribution ratio of the group in a certain gut microbiota. All data are expressed as means ± SEM. For normally distributed data (Firmicutes, Bacteroidetes, genera within the Ruminococcaceae family and Lachnospiraceae family, Lactobacillus_gasseri, Lactobacillus_murinus, Lactobacillus_reuteri), one-way ANOVA followed by Tukey’s test was used. For non-normally distributed data (Lactobacillus), Kruskal-Wallis test followed by non-parametric Wilcoxon rank-sum test was used. *p < 0.05, **p < 0.01 vs. the sham group; ^##^ *p* < 0.01 vs. the model group.

Given that the restoration of the gut microbiota played a key role in alleviating diseases (Ren et al., 2020; Zhu et al., 2021), we subsequently examined the effects of YQHG on gut microbiota in 5/6 nephrectomized rats. PCoA based on Bray-Curtis distance revealed that the gut microbial composition of the YQHG group was close to that of the sham group and distinct from that of the model group (Figure 3A). The community analysis at the phylum level showed that compared with the sham group and the YQHG group (Figure 3B), the gut microbial composition of the model group changed in certain bacteria, such as Firmicutes and Bacteroidetes (Figure 3C). In our study, 5/6 nephrectomy reduced the relative abundance of Firmicutes, while YQHG treatment increased its level to be close to that of sham-operated rats. Because Firmicutes constitutes the major SCFA-producing bacteria, our results might indicate an increase in SCFA production in YQHG-treated rats. Three families (e.g., Lachnospiraceae, Ruminococcaceae and Lactobacillaceae) are the major SCFA-producing taxa within Firmicutes phylum. Little is known about the functional capacity of these families individually; However, bacteria within these families had been found to be positively correlated with SCFA-related improvements in intestinal barrier integrity and function. The relative abundance of genera within Ruminococcaceae and Lachnospiraceae were not statistically different among the groups (Figure 3D). Within the Lactobacillaceae family, an increase in Lactobacillus was observed in the YQHG group (Figure 3E). To further understand the impact of YQHG on the Lactobacillus, we then conducted a detailed analysis of species within it. Among the species, Lactobacillus_gasseri was increased in abundance as a result of YQHG treatment (Figure 3E). Notably, the SCFA-producing bacteria improved by YQHG treatment were the dominant bacteria in the three groups, which were shown in Figure 3F.

**FIGURE 4:**
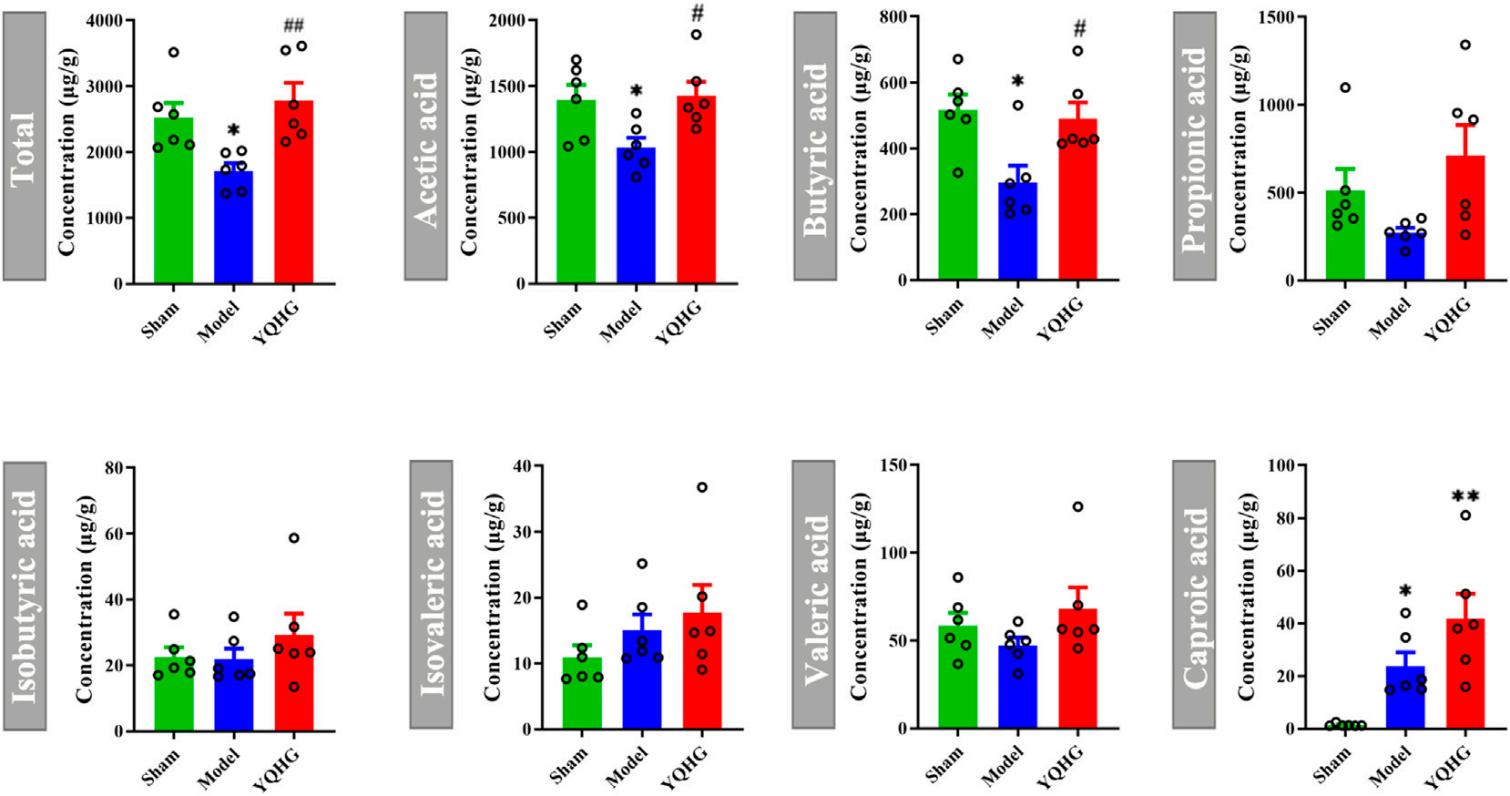
YQHG treatment regulates the concentrations of fecal SCFAs in 5/6 nephrectomized rats. All data are expressed as means ± SEM. For normally distributed data (Total, Acetic acid), one-way ANOVA followed by Tukey’s test was used. For non-normally distributed data (Butyric acid, Propionic acid, Isobutyric acid, Isovaleric acid, Valeric acid, Caproic acid), Kruskal-Wallis test followed by non-parametric Wilcoxon rank-sum test was used. *p < 0.05 vs the sham group; ^#^ *p* < 0.05, ^##^ *p* < 0.01 vs. the model group.

The restoration of SCFAs played a key role in improving the intestinal barrier to alleviate CKD (Knauf et al., 2019), we then considered whether YQHG affected SCFA concentrations in 5/6 nephrectomized rats. Our results showed that YQHG treatment significantly increased the concentrations of fecal SCFAs, especially the acetic acid and butyric acid (Figure 4). Taken together, these results demonstrated that YQHG treatment effectively reshaped the gut microbiota and increased the abundance of SCFA-producing bacteria, leading to increased fecal acetic acid and butyric acid concentrations in 5/6 nephrectomized rats.

### YQHG Improved Intestinal Barrier Integrity in 5/6 Nephrectomized Rats

**FIGURE 5:**
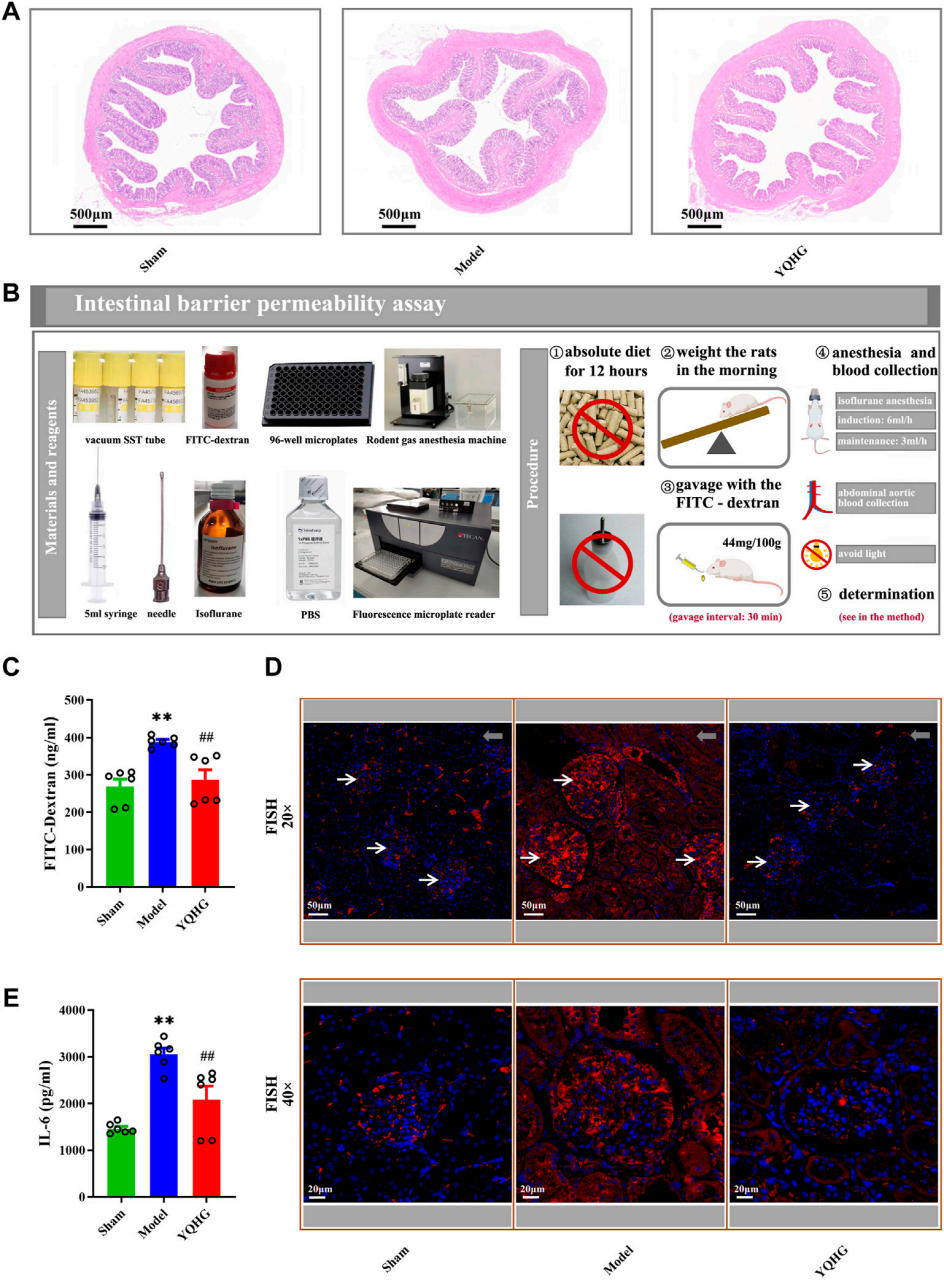
YQHG improves intestinal barrier integrity in 5/6 nephrectomized rats. **(A)** Representative images of H&E (2.0 × Magnification, Scale bar 500 μm) staining of colon tissues. **(B)** Details of the gut barrier permeability assay. **(C)** The concentration of FITC-dextran in serum was determined. **(D)** Representative images of FISH analysis (20 × Magnification, Scale bar 50 μm; 40 × Magnification, Scale bar 20 μm) in renal tissues. The red and blue fluorescent signals represent the probe and the nucleus, respectively. White arrows represent glomerulus, and gray arrows represent tubulointerstitium (20 × Magnification, Scale bar 50 μm). **(E)** The expression of IL-6 in renal tissues was determined. All data are expressed as means ± SEM. For non-normally distributed data (FITC-Dextran, IL-6), Kruskal-Wallis test followed by non-parametric Wilcoxon rank-sum test was used. **p < 0.01 vs. the sham group; ^##^ *p* < 0.01 vs. the model group.

The colon tissue of 5/6 nephrectomized rats was damaged as shown by HE staining, including obvious edema of the mucosa and submucosa, and distortion of the crypts. Specifically, widened openings and clubbed finger-like changes appeared in the crypts. However, these damage signs were attenuated by YQHG treatment (Figure 5A). In addition, the concentration of FITC-dextran in the model group was significantly higher than that in the sham group and the YQHG group. In contrast, with YQHG treatment, the permeability of intestinal barrier was significantly improved, characterized by decreased FITC-dextran concentration (Figures 5B,C). FISH is an efficient approach to localize the spatial distribution of bacteria in tissue samples. Compared with the sham group and YQHG group, the renal tissue of the model group had stronger bacterial signals (Figure 5D). Our previous study found that the key active compound quercetin in YQHG possessed a perfect binding conformation with IL-6 (Supplementary Figure S2). Moreover, researches have found that the expression of IL-6 is closely related to renal inflammation and fibrosis (Feigerlová and Battaglia-Hsu, 2017; Rops et al., 2018; Rocha et al., 2021). Thus, in this study, IL-6 was selected by us as a potential marker to evaluate renal inflammation. As expected, a marked decrease in IL-6 expression was observed in the YQHG group (Figure 5E). Taken together, our findings suggested that YQHG significantly improved intestinal barrier integrity and microbial translocation, ultimately contributing to reduced renal inflammation.

### Receiving Gut Microbiota From YQHG-Treated Rats Improved Renal Function and Fibrosis

In order to determine whether the therapeutic effects of YQHG on CKD were causally related to its regulation of the gut microbiota, we subsequently conducted a microbiota-transfer study (co-housing and FMT).

**FIGURE 6:**
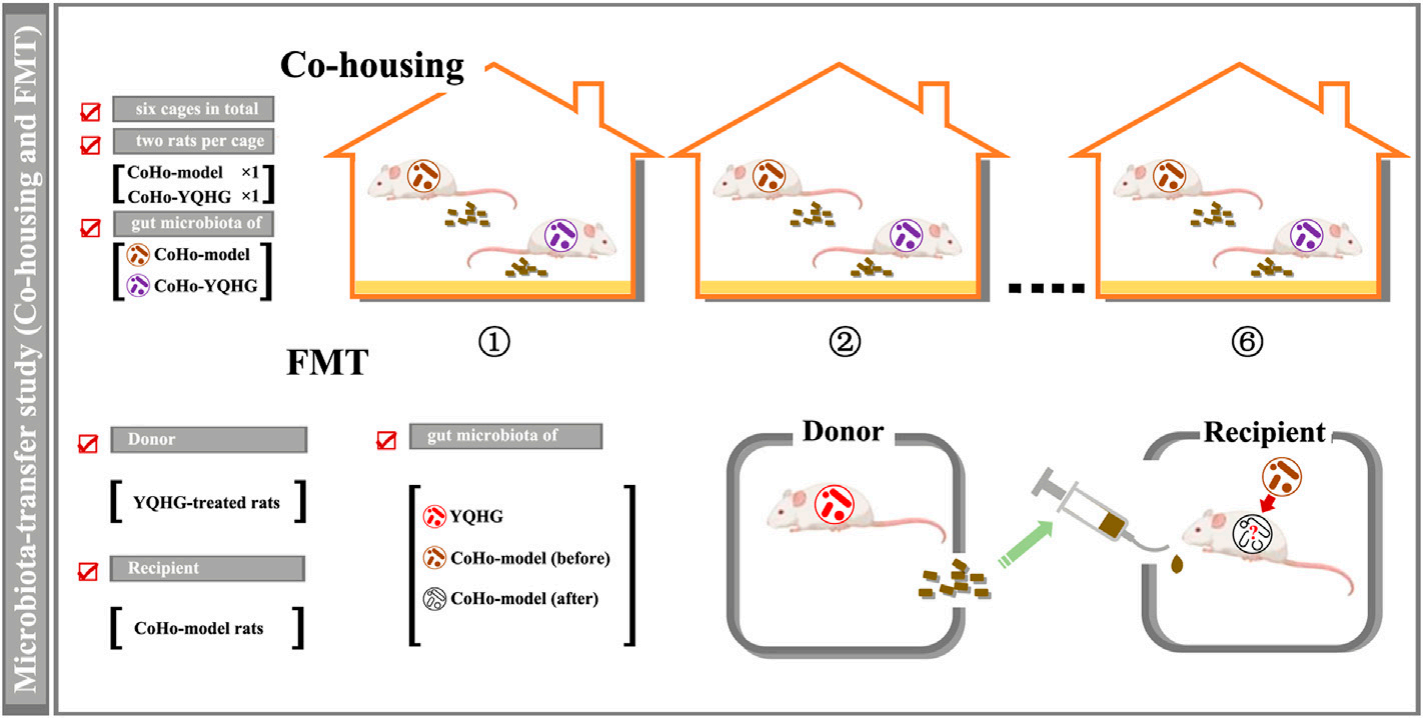
Design of microbiota-transfer study (Co-housing and FMT).

**FIGURE 7:**
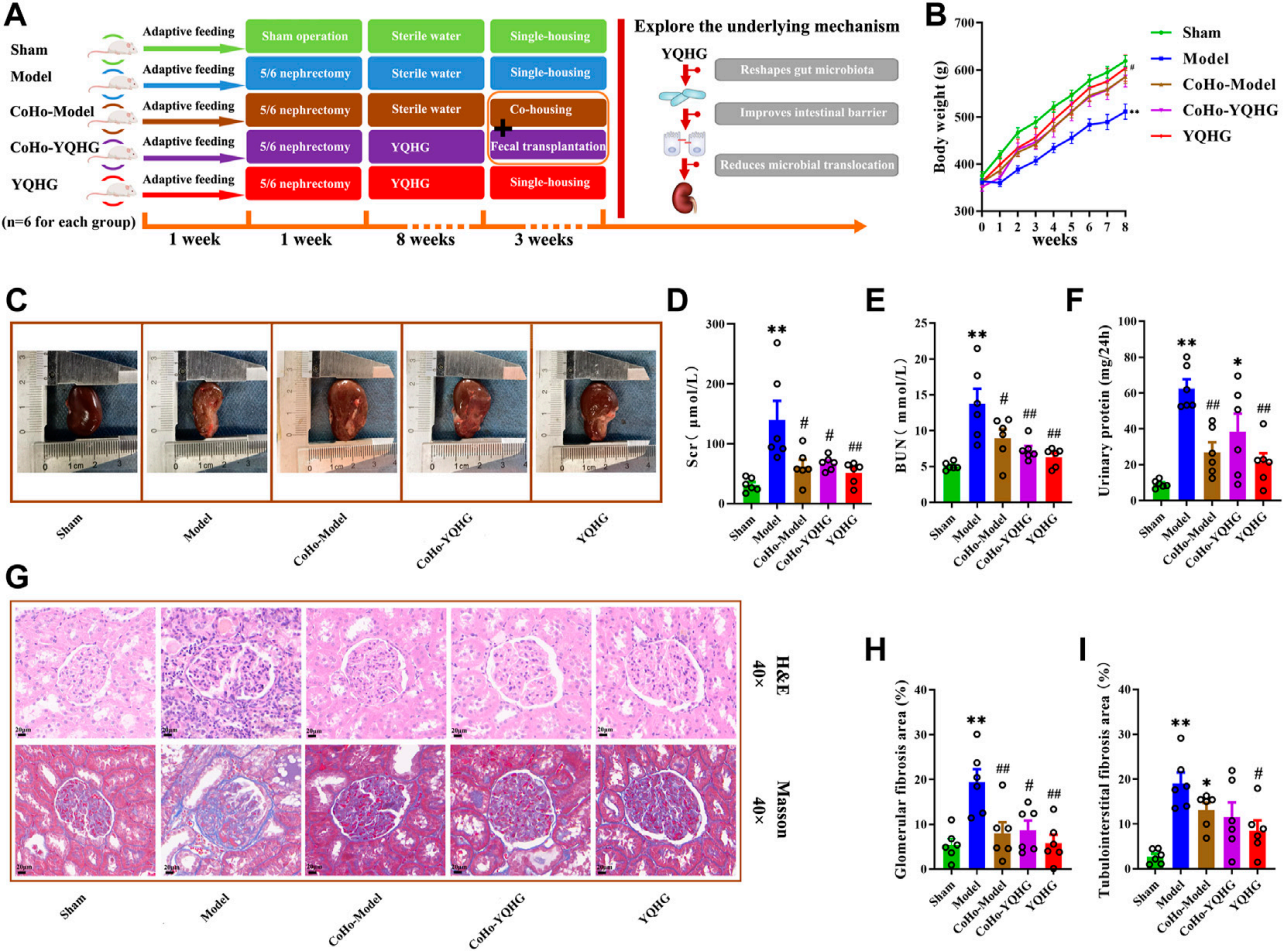
Receiving gut microbiota from YQHG-treated rats improves renal function and fibrosis. **(A)** Experimental design. **(B)** The body weight of the rats was measured. **(C)** The kidney of the rats was photographed. **(D–F)** Effects of fecal transfer (transplant) from YQHG-treated rats on Scr, BUN, and urinary protein levels. **(G)** Representative images of H&E (40 × Magnification, Scale bar 20 μm) staining and Masson (40 × Magnification, scale bar 20 μm) staining of renal tissues. **(H,I)** Quantitative analysis of glomerular fibrosis area and tubulointerstital fibrosis area based on Masson staining. All data are expressed as means ± SEM. For normally distributed data (body weight, Scr, BUN, urinary protein, glomerular fibrosis area, tubulointerstital fibrosis area), one-way ANOVA followed by Tukey’s test was used. *p < 0.05, **p < 0.01 vs. the sham group; ^#^ *p* < 0.05, ^##^ *p* < 0.01 vs. the model group.

A total of five groups were included in this part of the experiment, namely the sham group, the model group, the CoHo-model group, the CoHo-YQHG group, and the YQHG group (*n* = 6 for each group) (Figure 7A). Specifically, the 5/6 nephrectomized rats treated with YQHG for 8 weeks were randomly divided into YQHG group and CoHo-YQHG group. At the same time, the 5/6 nephrectomized rats treated with sterile water for 8 weeks was randomly divided into model group and CoHo-model group. Among them, the CoHo-model group and the CoHo-YQHG group mean that model rats co-housed with YQHG rats. Based on the fecal-eating characteristics of rodents, model rats could receive gut microbiota from YQHG rats. The cages were divided as follows:

1) For the co-housing groups: a total of six cages with two rats per cage (CoHo-model group × 1, CoHo-YQHG group × 1). During the co-housing period (3 weeks), FMT was performed on the CoHo-model group (Figure 6). Based on the microbiota-transfer strategy (Co-housing and FMT), the CoHo-model group received gut microbiota from the YQHG-treated rats.
2) For non-co-housing groups: ① Sham group (a total of two cages with three rats per cage). ② model group (a total of two cages with three rats per cage). ③ YQHG group (a total of two cages with three rats per cage).

After the microbiota-transfer study, rat samples (urine, feces, serum, plasma, renal and colon tissues) were collected for evaluation of disease manifestations and clinical parameters. As expected, YQHG treatment significantly delayed the progression of CKD in rats compared with the model group. Notably, compared with the model group, the rats in the CoHo-model group showed increased body weight and improved renal appearance (colour, capsule, border) (Figures 7B,C information of Quercetin). In addition, the Scr, BUN and urinary protein1 levels of the CoHo-model group were significantly lower than than those of the model group (Figures 7D–F). Histopathological analysis showed that the inflammatory infiltration, mesangial expansion, tubular atrophy and dilation, glomerular sclerosis, and interstitial fibrosis in the model group were significantly ameliorated in the CoHo-model group (Figure 7G). Quantitative analysis based on Masson staining showed that the area of glomerular fibrosis in the CoHo-model group was significantly lower than that in the model group (Figure 7H). In addition, although compared with the model group, the area of tubulointerstital fibrosis in the CoHo-model group was not significantly decreased, but there was a trend of improvement (Figure 7I).

### Receiving Gut Microbiota From YQHG-Treated Rats Altered Bacterial Communities and Increased SCFA Concentrations

**FIGURE 8:**
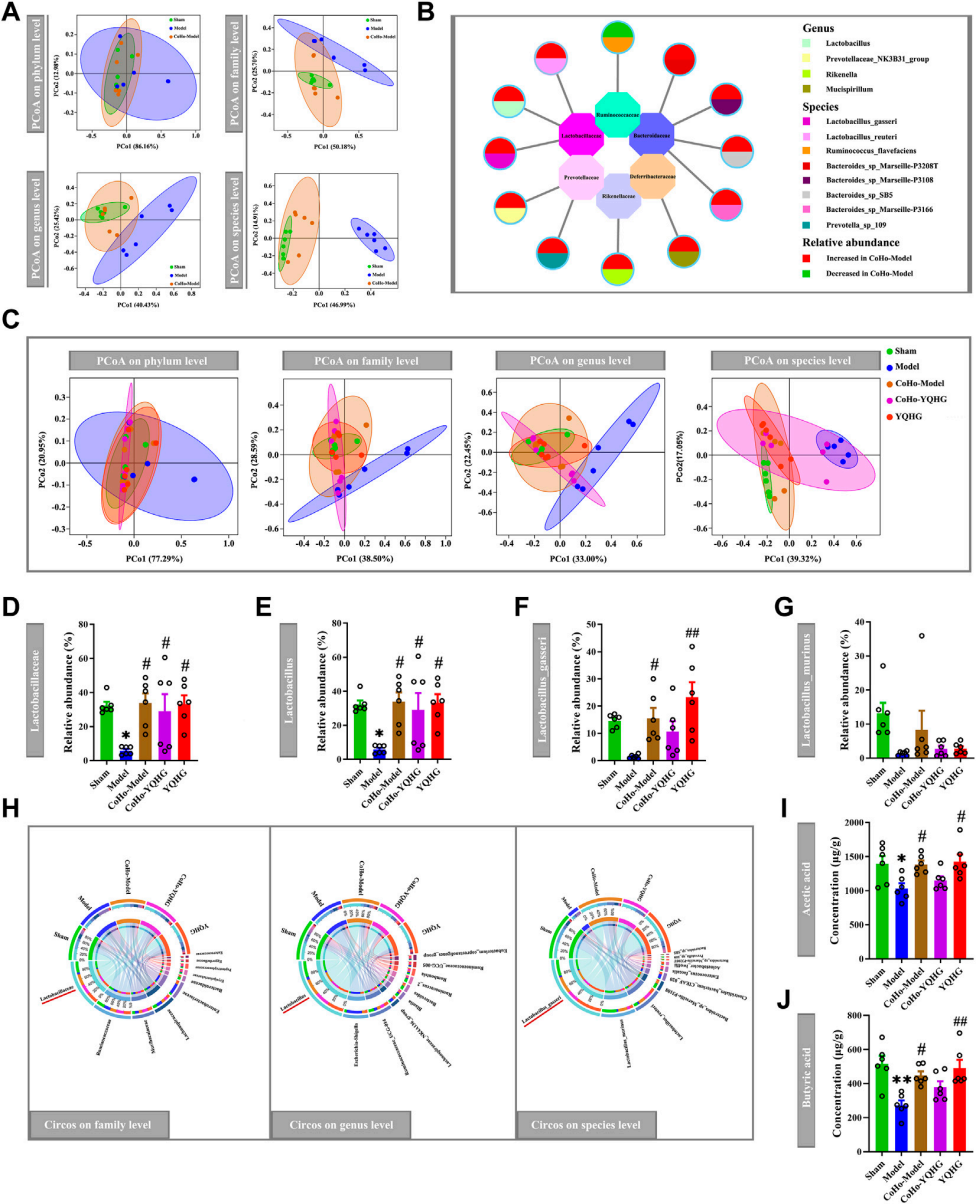
The alteration of gut microbiota and SCFA concentrations in co-housed rats. **(A,B)** Fecal transfer (transplant) from YQHG-treated rats modulates gut microbiota composition. PCoA based on Bray-Curtis distance in bacterial communities on four levels (phylum, family, genus, species) **(A)**. Differences in bacterial taxa between the model group and the CoHo-model group **(B)**. **(C–J)** The alteration of gut microbiota and SCFA concentrations in five groups. PCoA based on Bray-Curtis distance in bacterial communities on four levels (phylum, family, genus, species) **(C)**. Relative abundances of fecal Lactobacillaceae **(D)**, Lactobacillus **(E)**, Lactobacillus_gasseri **(F)**, and Lactobacillus_murinus **(G)**. Circos diagram of species-sample relationship on three levels (family, genus, species) **(H)**. The concentrations of fecal acetic acid **(I)** and butyric acid **(J)**. All data are expressed as means ± SEM. For normally distributed data (Lactobacillus_gasseri, Acetic acid), one-way ANOVA followed by Tukey’s test was used. For non-normally distributed data (Lactobacillaceae, Lactobacillus, Lactobacillus_murinus, Butyric acid), Kruskal-Wallis test followed by non-parametric Wilcoxon rank-sum test was used. *p < 0.05, **p < 0.001 vs. the sham group; ^#^ *p* < 0.05, ^##^ *p* < 0.01 vs. the model group.

In order to study the causal relationship between the gut microbiota and disease progression, we first realized the alteration of gut microbiota in co-housed rats based on the microbiota-transfer experiment. The sham group was used as the baseline, PCoA revealed that the gut microbial composition of the CoHo-model group was close to that of the sham group and distinct from that of the model group (Figure 8A). The different bacteria taxa between the model group and the CoHo-model group were shown in Figure 8B, Which were mainly distributed in three families (Lactobacillaceae, Bacteroidaceae, Prevotellaceae). Notably, 1/4 of these different bacteria belonged to the major SCFA-produding family (Lactobacillaceae). Our results showed that rats in the CoHo-model group had achieved gut microbiota transfer and remodeling. We further investigated the impact of microbiota-transfer study on the SCFA-producing bacteria and SCFA concentrations. PCoA revealed a distinct shift of bacteria communities in the model group compared with the other four groups (Figure 8C). Compared with the model group, the CoHo-model group demonstrated an increase in the relative abundance of SCFA-producing bacteria (Lactobacillaceae, Lactobacillus and Lactobacillus_gasseri) (Figures 8D–F). Notably, the SCFA-producing bacteria reversed by the CoHo-model group were the dominant bacteria in the five groups, as showen in Figure 8H.

In light of the discovery that receiving gut microbiota from YQHG-treated rats could remarkably increase the relative abundance of SCFA-producing bacteria, we subsequently evaluated the effect of microbiota-transfer (transplant) on fecal SCFA concentrations. Indeed, our results showed that the fecal acetic acid and butyric acid concentrations of the CoHo-model group were significantly higher than those of the model group (Figures 8I,J).

### Receiving Gut Microbiota From YQHG-Treated Rats Improved Intestinal Barrier Integrity

**FIGURE 9:**
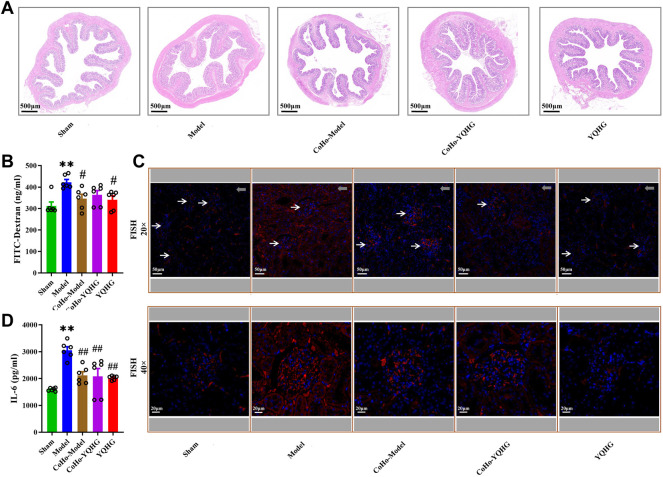
Receiving gut microbiota from YQHG-treated rats improves intestinal barrier integrity. **(A)** Representative images of H&E (2.0 × Magnification, Scale bar 500 μm) staining of colon tissues. **(B)** The concentration of FITC-dextran in serum was determined. **(C)** Representative images of FISH analysis (20 × Magnification, Scale bar 50 μm; 40 × Magnification, Scale bar 20 μm) in renal tissues. The red and blue fluorescent signals represent the probe and the nucleus, respectively. White arrows represent glomerulus, and gray arrows represent tubulointerstitium (20 × Magnification, Scale bar 50 μm). **(D)** The expression of IL-6 in renal tissue was determined. All data are expressed as means ± SEM. For non-normally distributed data (FITC-Dextran, IL-6), Kruskal-Wallis test followed by non-parametric Wilcoxon rank-sum test was used. **p < 0.01 vs. the sham group; ^*#*^ *p* < 0.05, ^##^ *p* < 0.01 vs. the model group.

To gain further insight into the mechanisms by which co-housing provides protection to the kidneys, we subsequently analyzed the alteration of gut barrier integrity in co-housed rats. HE staining of the colon tissue showed that mucosal and submucosa edema, and crypt distortion were significantly improved in the CoHo-model group compared with the model group (Figure 9A). Furthermore, the decreased permeability of intestinal barrier and controlled microbial translocation were observed in the CoHo-model group (Figures 9B–D). Finally, compared with the model group, renal inflammation was alleviated in the CoHo-model group, which was characterized by decreased IL-6 expression (Figure 9E). Thus, our results suggested that intestinal barrier protection provided by co-housing was causally related to the mediating effects of gut microbiota, especially SCFA-producing bacteria.

## Discussion

YQHG is a prescription based on the pathogenesis of renal disease in TCM, and it has been used in clinical practice for a long time. Our previous study showed that YQHG could ameliorate renal dysfunction by regulating autophagy, apoptosis and inflammatory response (Liu et al., 2016). However, the potential mechanism of YQHG in treating CKD based on the gut microbial ecosystem has never been explored. Our present study clearly indicated that YQHG treatment significantly prevented the progression of CKD, which was characterized by the increased body weight, improved renal appearance and function, reduced tissue damage in 5/6 nephrectomized rats. Regardless of the cause, renal fibrosis is a common result of most progressive renal diseases and is closely related to worsening renal function. Importantly, we demonstrated that 5/6 nephrectomized rats treated with YQHG showed a significant improvement in renal fibrosis, as evidenced by the reduction in the area of glomerular and tubulointerstitial fibrosis. Of note, we found that YQHG played an important role in regulating bacterial communities, especially increasing the relative abundance of SCFA-producing bacteria, which in turn led to the improved SCFA concentrations and intestinal barrier integrity. Ultimately, controlled microbial translocation contributed to the alleviation of renal inflammation. Our microbiota-transfer study (co-housing and FMT) further supported that the protective effects of YQHG against CKD depended on its modulation of gut microbiota. Therefore, the present study not only provides a new perspective on the pathogenesis of CKD, but also confirms that YQHG has a potential therapeutic value for CKD by targeting the gut microbiota.

CKD is characterized by increased morbidity and mortality. Although the underlying mechanism of CKD is still elusive, evidence has suggested that part of its pathogenesis is attributed to the imbalance of the gut microbial ecosystem (Meijers et al., 2019). In this study, a CKD rat model was constructed by 5/6 nephrectomy. Removal of 5/6 renal parenchyma effectively reduces the number of nephrons, which in turn leads to the elevated perfusion, filtration and pressure of the remaining nephrons. Persistent overload leads to 1/6 remnant renal parenchyma unable to maintain homeostasis, which ultimately contributes to the gradual loss of renal function (Kujal and Vernerová, 2008). At present, the 5/6 nephrectomy model has been recognized as the classical model most similar to human CKD. It has been widely used to study the therapeutic effect of drugs on CKD (Mizukami et al., 2019; López-Baltanás et al., 2021; Zhou et al., 2021). Moreover, the 5/6 nephrectomy model also show significant gut microbiota dysbiosis (Liu et al., 2018; Feng et al., 2019), and this model may be suitable in this study. Our current study showed that significant changes in the bacterial community were observed in 5/6 nephrectomized rats, which was consistent with previous studies (Nishiyama et al., 2019; Ji et al., 2021). Of note, YQHG treatment conferred a profound protection of gut microbiota, preventing the imbalance of bacterial community and the impairment of SCFA-producing bacteria in 5/6 nephrectomized rats. Specifically, bacteria that contain SCFA-producing taxa (e.g., members of the Lactobacillaceae) were reduced in relative abundance as a result of rats with 5/6 nephrectomy. These results implied that the reduced SCFA-producing bacteria constituted part of the pathogenesis of CKD, and it may become a promising therapeutic target for the drugs against CKD. To support this notion, our study demonstrated that within the Lactobacillaceae family, the relative abundance of Lactobacillus and Lactobacillus_gasseri was significantly restored by YQHG treatment. In concert with the observation of increased SCFA-producing taxa, we found that prominent SCFAs in feces, including acetic acid and butyric acid were markedly higher in rats with YQHG treatment.

An accumulating body of evidence now suggests that impaired intestinal barrier contributes to many intestinal or extra-intestinal diseases, including inflammatory bowel diseases (IBDs) (Cheng et al., 2021), systemic lupus erythematosus (SLE) (Pan et al., 2021), obesity, etc (Kang et al., 2021). The latest study has found that the impaired intestinal barrier is also existed in CKD, which is known as leaky gut. It is characterized by increased permeability of intestinal barrier (Meijers et al., 2018). The underlying mechanisms of intestinal barrier damage related to gut microbiota imbalance in CKD are as follows: ①The blood urea concentration in CKD patients is significantly increased, which promotes the proliferation of urease-producing bacteria after dispersing into the intestinal lumen. Urea is hydrolyzed by urease to produce ammonia, which results in increased ammonia production in the intestinal lumen due to urease imbalance. As a result, it causes increased intestinal pH, mucosal damage, and structural damage to the intestinal wall, ultimately leading to the increased permeability of intestinal barrier (Vaziri et al., 2013b). ②The imbalance of SCFA-producing bacteria leads to a decrease in the SCFAs concentrations, which greatly reduces the nutrition and energy sources of colon tissue. In theory, it can aggravate intestinal barrier damage (Vos et al., 2022). ③Intestinal barrier damage stimulates leukocyte infiltration, resulting in local inflammation and related pro-inflammatory cytokines that can induce tight junction protein endocytosis, further leading to increased permeability of intestinal barrier (Al-Sadi et al., 2009).

Increased intestinal permeability may lead to the translocation of bacterial components from the intestinal lumen to systemic circulation, ultimately contributing to aggravated renal inflammation (Lau et al., 2015). These results suggest that effective improvement of intestinal barrier may be the key to alleviating the progression of CKD. The values of SCFAs in maintaining intestinal homeostasis had been widely explored (Felizardo et al., 2019). Higher concentrations of SCFAs decrease luminal PH, which brings perfect environment for the production of acetic acid, propionic acid and butyric acid (Morrison and Preston, 2016). Acetic acid, which is the most abundant SCFA, is a vital co-factor for bacteria growth (Duncan et al., 2004). Moreover, propionic acid and butyric acid are energy sources for colonocytes, and these SCFAs may enhance intestinal health by reducing the permeability of the intestinal barrier (Pace et al., 2021; Tang et al., 2022). Evidence showed that the concentrations of SCFAs, especially acetic acid and butyric acid, were significantly reduced in CKD. However, improved intestinal barrier and renal function were observed through the supplementation of SCFAs, especially butyric acid (Deng et al., 2021). Previous studies indicated that the alleviation of renal dysfunction was related to the improved SCFA concentrations and intestinal barrier.

Our subsequent research further supported this notion. First, it was consistent with the fact that the reduced total SCFA concentrations were observed in 5/6 nephrectomized rats. However, it was worth noting that YQHG treatment significantly restored the concentrations of fecal total SCFAs, especially acetic acid and butyric acid. Furthermore, FD-4 experiment suggested that the intestinal permeability of rats with YQHG treatment was significantly improved. Moreover, by FISH analysis, we found that the bacterial signals in the renal tissue of 5/6 nephrectomized rats was significantly higher than that of rats with YQHG treatment. This result indirectly supported the view that YQHG could improve the intestinal barrier and control the influx of bacterial components into the kidney via the systemic circulation. Our previous study found that the key active compound quercetin in YQHG possessed a good binding conformation with IL-6. In addition, researches reported that the reduction of IL-6 expression may contribute to alleviate tissue inflammation and fibrosis in CKD rats (Bian et al., 2019). Therefore, in the end, we explored the improvement of renal inflammation by targeting IL-6. Our current study found that YQHG treatment decreased the expression of IL-6 in renal tissue, which was consistent with previous results.

To further confirm the importance of gut microbiota for YQHG in the treatment of CKD, microbiota-transfer study (co-housing and FMT) was used to reshape the bacterial community of co-housed animals. Impressively, the kidneys of the rats in the CoHo-model group was deeply protected after the microbial transfer, which was characterized by the alleviated renal inflammation, fibrosis and dysfunction. The results showed that the protective effect of YQHG was partly attributed to the mediation of the gut microbiota, especially the SCFA-producing bacteria. Future research may be needed to investigate how YQHG supports the survival or expansion of SCFA-producing bacteria.

In summary, our data identified YQHG as an important exogenous regulator of crosstalk between the gut microbial ecosystem and CKD. Specifically, the improved SCFA-producing bacteria and intestinal barrier were critically involved in the therapeutic effect of YQHG on CKD. Our current findings propose a microbiota-targeted intervention and indicate that YQHG may become a novel promising treatment for CKD.

## Limitation

1) In addition to constructing a surgical-induced model, a second non-surgical experimental model of CKD (such as the adenine-induced CKD rat model) should be included to rule out model-specific in subsequent experiments. (2) Given known variability in surgically induced CKD, it is necessary to check for kidney function measures prior to initiation of the intervention in our subsequent experiments. (3) The gut-derived uremic toxins (e.g., IS, pCS). are closely associated with CKD. In the context of CKD, the increased synthesis of IS and pCS (the shift in the metabolic pattern of gut microbiota leads to the increase of precursors) and the decreased excretion (high protein affinity and decreased renal tubular secretory function) further promote the progression of CKD (Yang et al., 2018; Huang et al., 2020). The detection of those uremic toxins should be included in our follow-up studies.

## Data Availability

The datasets presented in this study can be found in online repositories. The names of the repository/repositories and accession number(s) can be found below: National Center for Biotechnology Information (NCBI) BioProject database under accession number PRJNA817737.

## Supplementary Material

Supplementary Figure S1 Fingerprint of YQHG based on UHPLC-MS approach.

Supplementary Figure S2 The information of Quercetin and Kaemferol. **(A)** Extracted and amplified the peak graph for the two components. **(B)** The secondary mass spectrometry information of the two components.

Supplementary Figure S3 The binding mode of IL-6 protein with Quercetin. **(A)** The 3D structure of complex. **(B)** The surface of active site. **(C)** The detail binding mode of complex. The backbone of protein is rendered in tube and colored in green. Compound is rendering by yellow. Yellow dash represents hydrogen bond distance or π-stacking.
