# Supplementary figures and images for: Yishen Qingli Heluo Granule Ameliorates Renal Dysfunction in 5/6 Nephrectomized Rats by Targeting Gut Microbiota and Intestinal Barrier Integrity
Source: Front Pharmacol. 2022 Jun 22;13:858881. doi: 10.3389/fphar.2022.858881 (PMC9258868; doi:10.3389/fphar.2022.858881)

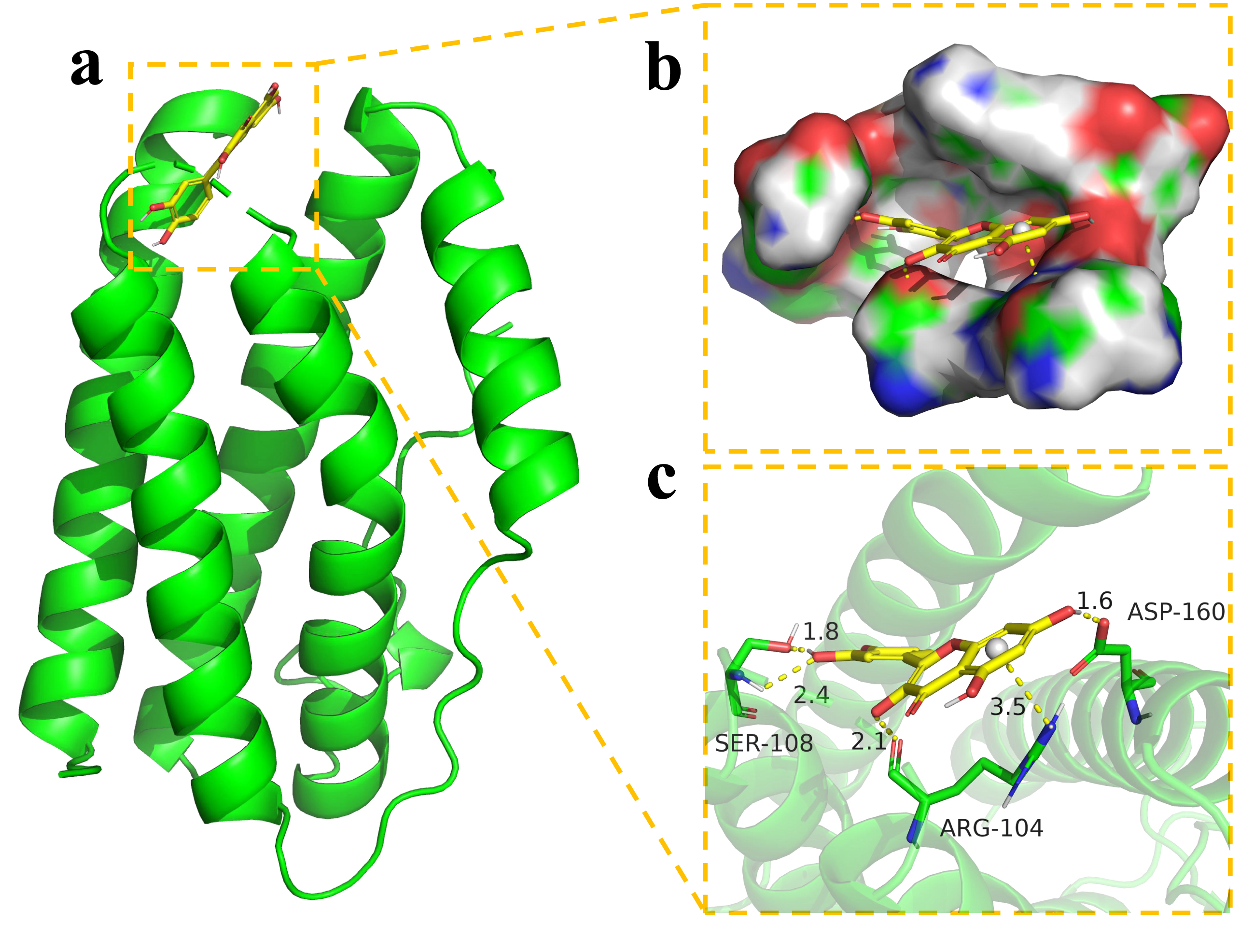

Supplement: Supplementary file 1 [file Image3.JPEG]

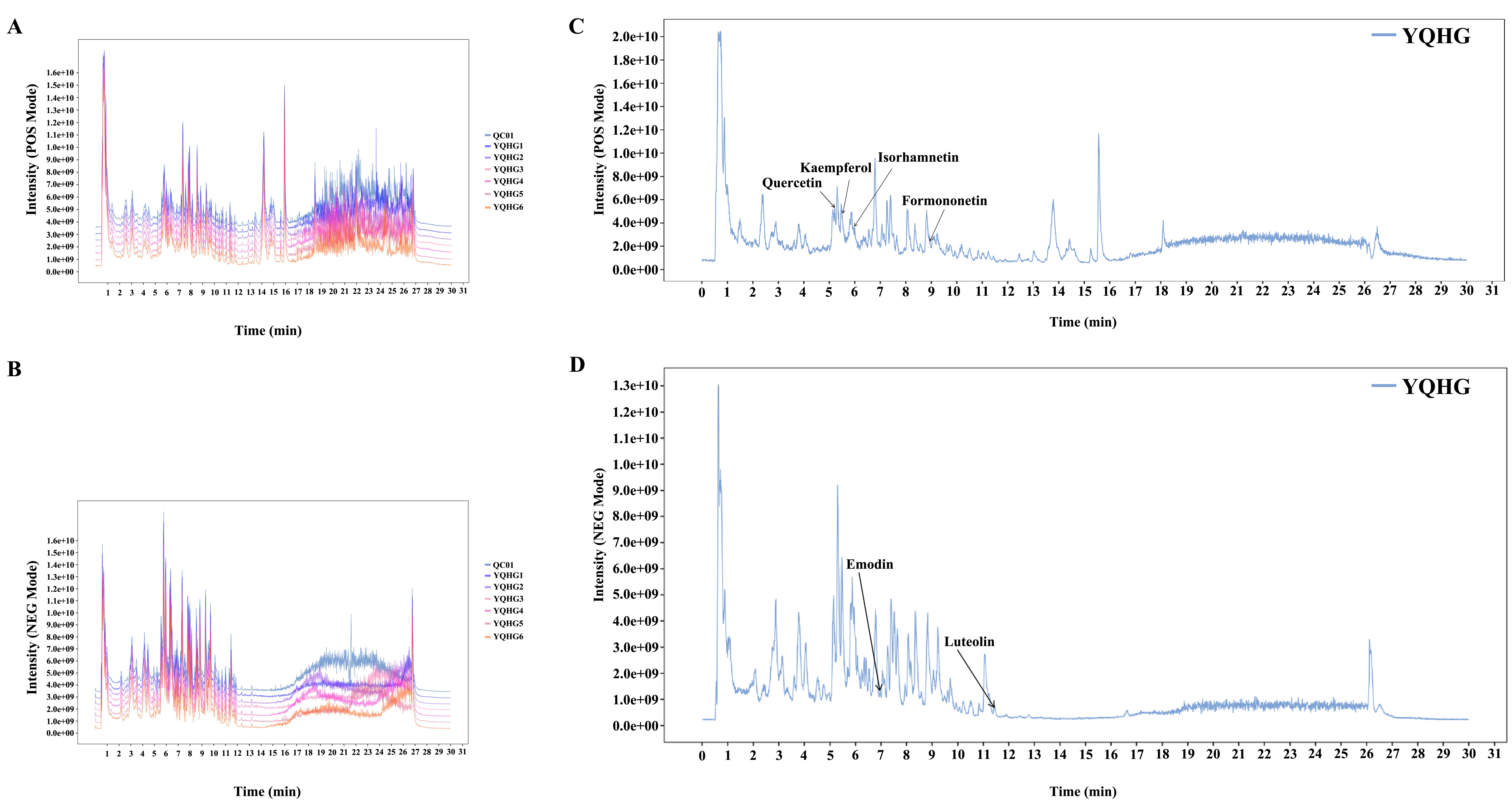

Supplement: Supplementary file 2 [file Image1.JPEG]

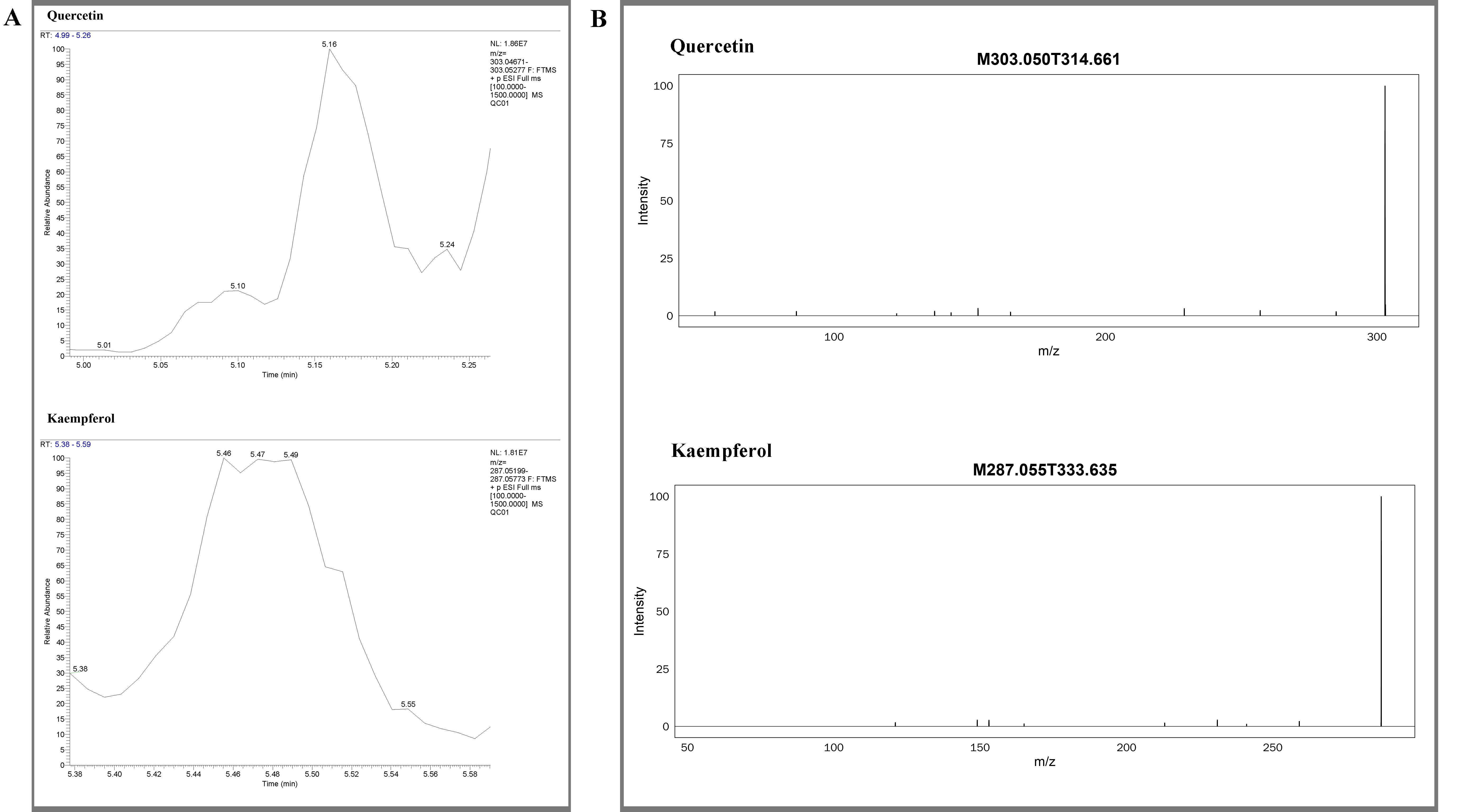

Supplement: Supplementary file 3 [file Image2.JPEG]
