# Supplementary material for: Yishen Qingli Heluo Granule Ameliorates Renal Dysfunction in 5/6 Nephrectomized Rats by Targeting Gut Microbiota and Intestinal Barrier Integrity
Source: Front Pharmacol. 2022 Jun 22;13:858881. doi: 10.3389/fphar.2022.858881 (PMC9258868; doi:10.3389/fphar.2022.858881)
Supplement: Supplementary file 4 [file Table1.DOC]

**TABLE S1 The information of the six compounds.**

| **Mode** | **Compound** | **InChIKey** | **Formula** | **mzmed** | **rtmed** | **relative abundance (peak area)** |
| --- | --- | --- | --- | --- | --- | --- |
| POS | Quercetin | REFJWTPEDVJJIY-UHFFFAOYSA-N | C15H10O7 | 303.0497413 | 314.661 | 79270141.366 |
|  | Kaempferol | IYRMWMYZSQPJKC-UHFFFAOYSA-N | C15H10O6 | 287.0548686 | 333.6355 | 101531939.282 |
|  | Isorhamnetin | IZQSVPBOUDKVDZ-UHFFFAOYSA-N | C16H12O7 | 317.0655643 | 354.754 | 20461022.384 |
|  | Formononetin | HKQYGTCOTHHOMP-UHFFFAOYSA-N | C16H12O4 | 291.0632803 | 537.432 | 38913816.291 |
| NEG | Luteolin | IQPNAANSBPBGFQ-UHFFFAOYSA-N | C15H10O6 | 285.0409597 | 682.958 | 7592370.427 |
|  | Emodin | RHMXXJGYXNZAPX-UHFFFAOYSA-N | C15H10O5 | 269.0458669 | 418.395 | 11685385.472 |
